# Photocatalytic production and biological activity of D-arabino-1,4-lactone from D-fructose

**DOI:** 10.1038/s41598-024-84921-z

**Published:** 2025-01-11

**Authors:** Sho Usuki, Pratiksha Babgonda Patil, Tiangao Jiang, Naoko Taki, Yuma Uesaka, Haru Togawa, Sanjay S. Latthe, Shanhu Liu, Kenji Yamatoya, Kazuya Nakata

**Affiliations:** 1 Graduate School of Bio-Applications and Systems Engineering, Tokyo University of Agriculture and Technology, 2-24-16 Naka-cho, Koganei, Tokyo, 184-0012 Japan; 2 Vivekanand College, C.S. No 2130 E ward, Tarabai Park, Kolhapur, 416 003 Maharashtra India; 3 Henan Joint International Research Laboratory of Environmental Pollution Control Materials, Henan Key Laboratory of Polyoxometalate Chemistry, College of Chemistry and Chemical Engineering, Henan University, Kaifeng, 475004 PR China; 4 Laboratory of Genomic Function Engineering, Department of Life Sciences, School of Agriculture, Meiji University, 1-1-1 Higashimita, Tama-ward, Kawasaki, 214-8571 Kanagawa Japan

**Keywords:** Photocatalytic conversion, D-arabino-1,4-lactone, Biomass valorization, Prebiotic compounds, TiO_2_, Heterogeneous catalysis, Photocatalysis

## Abstract

Lactones play crucial roles in various fields, such as pharmaceuticals, food, and materials science, due to their unique structures and diverse biological activities. However, certain lactones are difficult to obtain in large quantities from natural sources, necessitating their synthesis to study their properties and potential. In this study, we investigated the photocatalytic conversion of D-fructose, a biomass-derived and naturally abundant sugar, using a TiO_2_ photocatalyst under light irradiation in ambient conditions. The resulting products were identified using HPLC, LCMS, MALDI TOF MS, and ^1^H NMR. The results confirmed the successful production of D-arabino-1,4-lactone as a key product, along with the formation of other valuable compounds, including rare sugars such as erythrose and glyceraldehyde. Analysis of the reaction mechanism revealed that D-arabino-1,4-lactone can be directly produced by the α scission (C_1_-C_2_ position cleavage) of D-fructose. Furthermore, erythrose and glyceraldehyde, as rare sugars, can be produced from the decomposition of D-arabino-1,4-lactone, which means that D-arabino-1,4-lactone can be used as a source of rare sugars. Furthermore, to investigate the biological activity of D-arabino-1,4-lactone, it was administered to *Bifidobacterium*. The results showed that *Bifidobacterium* proliferated and produced more lactic acid than when cultured in a medium without D-arabino-1,4-lactone, suggesting that *Bifidobacterium* can utilize D-arabino-1,4-lactone.

**Supplementary Information:**

The online version contains supplementary material available at 10.1038/s41598-024-84921-z.

Biomass is gaining attention as a renewable resource in the pursuit of a sustainable society. Bio-based materials can be converted into fuels^1^, pharmaceutical raw materials^2^, and other high-value compounds^3,4^, making it promising for use in a wide range of fields^5,6^, including the chemical and pharmaceutical industries^7,8^. Furthermore, plant matter has the potential to contribute to mitigating global warming by acting as a carbon dioxide sink^9,10^. These characteristics have led to increased interest in the research and development of biomass and its derivatives, accelerating efforts towards contributing to a sustainable society and reducing environmental impact^11,12^. The useful substances derived from biomass are diverse, ranging from fuels to chemical raw materials, food, pharmaceuticals, and cosmetics^13–15^, with expected applications in areas such as ethanol^16^, biodiesel^17^, lactic acid^18^, and aromatic compounds^19^. These substances can provide environmentally friendly and sustainable alternatives to conventional products that depend on fossil fuels.

Lactones are cyclic esters formed by the intramolecular esterification of hydroxy carboxylic acids and play important roles in various fields because of their unique structural characteristics and diverse biological activities^20^. For example, δ-decalactone is used as a food flavor^21^, whereas gluconolactone is an important component in pharmaceutical synthesis^22^. γ-Butyrolactone is widely used as a solvent and precursor in chemical synthesis^23^, and ε-caprolactone plays a crucial role as a monomer for the biodegradable polymer polycaprolactone (PCL)^24,25^. Moreover, the biodegradability of many lactones is an important characteristic that contributes to reducing environmental burden and developing sustainable products^26,27^. This feature further enhances the appeal of lactones, especially for the development of environmentally conscious products and materials. As seen in the example of PCL, lactones and their derivatives serve as the foundation for developing innovative materials that combine biocompatibility and environmental compatibility^28^. However, certain lactones are difficult to obtain in large quantities from natural sources. Therefore, there is an urgent need to develop technologies for synthesizing these compounds in an environmentally friendly manner to study their properties and potential. The development of sustainable processes for lactone production not only addresses increasing demand but also contributes to more environmentally responsible and economically viable industrial practices.

Lactone synthesis using photocatalysts is a promising approach to address this challenge. Photocatalysts are a group of substances that oxidize or reduce substrates near their surface under light irradiation in ambient conditions^29,30^. Kaplaneris et al. synthesized lactones from alcohols using photoorganocatalytic synthesis with visible light^31^, while Triandafillidi et al. converted alkenes to γ-lactones using a Ru(bpy)_3_Cl_2_ photocatalyst^32^. Wei et al. reported the formation of γ-lactones by reacting styrene and R-bromoesters using the visible light photoredox catalyst *fac*-Ir(ppy)_3_^33^, and Özgen et al. achieved the synthesis of chiral γ-lactones from aldehydes and olefins using a combination of a tetrabutylammonium decatungstate photocatalyst and a biocatalyst^34^.

These previous studies have demonstrated the potential of environmentally friendly lactone synthesis using photocatalysts. However, to realize a sustainable society, not only environmentally friendly reaction processes but also the use of renewable resources as starting materials are necessary. Despite this necessity, research on methods for directly synthesizing lactones from renewable resources such as biomass has been scarce.

In this study, we demonstrated the direct conversion of D-fructose, a biomass-derived compound, to produce D-arabino-1,4-lactone as a lactone using a TiO_2_ photocatalyst under light irradiation in ambient condition. The products were analyzed by HPLC, LCMS, MALDI TOF MS, and ^1^H NMR. Additionally, we examined whether the D-arabino-1,4-lactone produced could be metabolized by bacteria beneficial to the human body. This study aims to elucidate the specific reaction pathways and intermediates involved in the photocatalytic conversion of D-fructose, and to explore the potential biological activities of the produced lactone, particularly focusing on their effects on beneficial gut bacteria.

## Methods

### TiO_2_ treatment of D-fructose and D-arabino-1,4-lactone

An aqueous solution (100 mL) containing 60 mmol L^− 1^ D-fructose (FUJIFILM Wako Pure Chemical Corp.) was mixed with 35 mg TiO_2_ photocatalyst powder (P25, Degussa). The obtained suspension was stirred at 350 rpm for 15 min in the dark using a magnetic stirrer, and then irradiated with UV light at an intensity of 10 mW cm^− 2^ by a Hg-Xe lamp (UV-7, U-VIX) at 20 ºC under atmospheric pressure for 144 h. The sample was collected at each 24 h. Afterward, the TiO_2_ powder was removed from the suspension using a 0.22 μm nylon filter to obtain the filtrate. Similarly, 2 mg of TiO_2_ photocatalyst powder was added to 20 mL of an aqueous solution containing 20 mmol L^− 1^ D-arabino-1,4-lactone (Sigma Aldrich). The obtained suspension was stirred at 350 rpm for 15 min in the dark, and then irradiated with UV light at an intensity of 5 mW cm^− 2^ at 20 °C under atmospheric pressure. Subsequently, the TiO_2_ powder was removed from the suspension using a 0.22 μm nylon filter to obtain the filtrate.

### Product analysis

The analysis of the starting materials D-fructose and D-arabino-1,4-lactone in the reaction solution was performed using HPLC (Shimadzu Corp.) equipped with a refractive index detector (RI). The analytical column used was Sugar-D (250 mm × 4.6 mm, 5 μm particle size, COSMOSIL), and the mobile phase was 75% acetonitrile/25% water (v/v) at a flow rate of 1.0 mL min^− 1^ at 30 °C. The concentrations of D-fructose and D-arabino-1,4-lactone were calculated using the calibration curves. The reaction products arabinose, erythrose, and glyceraldehyde in the reaction solution were measured at 305 nm using HPLC equipped with a UV-VIS detector (UV-VIS). Before measurement, arabinose, erythrose, and glyceraldehyde were derivatized with p-aminobenzoic acid ethyl ester (ABEE). The procedure was as follows: An ABEE solution was prepared by mixing 332.4 mg ABEE, 31.7 mg sodium cyanoborohydride, 386.4 µL acetic acid, and 3.6 mL methanol. Then, 10 µL of the reaction solution at each irradiation time was mixed with 40 µL of the ABEE solution and vortexed for 15 s. After centrifugation for 2 min, the mixture was heated to 80 ºC. After 1 h, the mixture was cooled to room temperature and centrifuged for 2 min. Water (200 µL) and chloroform (200 µL) were then added to the mixture. After vortexing for 1 min, the mixture was centrifuged for 2 min. Subsequently, 150 µL of the upper aqueous layer of the two-layer liquid of water and chloroform was collected, and 300 µL of water was added to the aqueous solution. After vortexing for 1 min, the mixture was centrifuged for 2 min. This solution was used for HPLC analysis. The analytical column used was CAPCELL PAK C18 (150 mm × 4.6 mm, 3 μm particle size, OSAKA SODA), and the mobile phase was an 87% ammonium acetate solution (20 mmol L^− 1^)/ 13% acetonitrile (v/v) at a flow rate of 1.0 mL min^− 1^ at 40 °C. The concentrations of arabinose, erythrose, and glyceraldehyde were calculated using calibration curves. The molecular weights of the reaction products, arabinose, erythrose, and glyceraldehyde, were confirmed using LCMS (LCMS8050, Shimadzu Corp.). The analytical column used was CAPCELL PAK C18 (150 mm × 2.0 mm, 3 μm particle size, OSAKA SODA), and the mobile phase was 87% ammonium acetate solution (20 mmol L^− 1^)/13% acetonitrile (v/v) at a flow rate of 0.2 mL min^− 1^ at 40 ºC. The positive ESI mode was used.

Formaldehyde analysis was performed by measuring at 365 nm using the UV-VIS detector of HPLC. Before measurement, the products were derivatized with 2,4-dinitrophenylhydrazine (DNPH). The procedure was as follows: 30 mg of DNPH, 30 mL of methanol, 1.5 mL of hydrochloric acid, and 13.5 mL of ultrapure water were mixed by sonication for 10 min. After mixing, the solution was stored in the dark as a DNPH labeling reagent. The reaction solution was then diluted 50-fold with ultrapure water so that the concentration of formaldehyde was below 2 mM. 4 mL of the DNPH labeling reagent and 1 mL of the diluted solution were mixed and allowed to stand at 40 ºC for 10 min. After that, the mixture was left to stand in the dark at room temperature for 2 h. This solution was then used for HPLC analysis. The analytical column used was a Luna C18(2) (250 mm×4.6 mm I.D., 5 μm particle size, Shimadzu), and the mobile phase was 60% acetonitrile/ 40% water (v/v) at a flow rate of 1.0 mL min^− 1^ at 40 °C. The product concentration was calculated using a calibration curve. The molecular weight of formaldehyde was confirmed using LCMS (LCMS8050, Shimadzu Corp.). The analytical column used was Shim-pack VP-ODS (250 mm × 2.0 mm, 5 μm particle size, Shimadzu) with, and the mobile phase was 60% acetonitrile/40% water (v/v) at a flow rate of 0.2 mL min^− 1^ at 40 ºC. The positive ESI mode was used.

Formic acid and acetic acid analyses were performed by measuring at 210 nm using the UV-VIS detector of HPLC. Before the measurement, 2.5 µL of 1 mol/L sulfuric acid was added to 0.9975 mL of the solution so that the concentration of sulfuric acid was 2.5 mmol/L. This solution was used for HPLC analysis. The analytical column used was Rezex ROA-Organic Acid H+ (8%) (300 × 7.8 mm, 5 μm particle size, Phenomenex), and the mobile phase was 2.5 mmol/L sulfuric acid at a flow rate of 0.5 mL min^− 1^ at 60 ºC. The concentrations of formic and acetic acids were calculated from the calibration curves.

### Isolation of products

To isolate D-arabino-1,4-lactone for MALDI TOF MS and ^1^H NMR measurements, the following experiments were conducted. The experiments were performed under the same conditions as the TiO_2_ treatment of D-fructose described above. After 144 h of UV irradiation, all the reaction solutions were filtered through a 0.22 μm nylon filter to remove TiO_2_ from the suspension. Multiple batches of the reaction solution were collected and concentrated using a rotary evaporator. D-arabino-1,4-lactone was isolated using HPLC. The column used was Sugar-D (250 mm × 4.6 mm, 5 μm particle size, COSMOSIL), and the mobile phase was 75% acetonitrile/25% water (v/v) at a flow rate of 1.0 mL min^− 1^ at 30 ºC. Erythrose and glyceraldehyde were labeled with ABEE using the concentrated solution and then isolated by HPLC. The column used was a CAPCELL PAK C18 (150 mm × 4.6 mm, 3 μm particle size, SHISEDO), and the mobile phase was 87% ammonium acetate solution (20 mmol L^− 1^) and 13% acetonitrile (v/v) at a flow rate of 1.0 mL min^− 1^ at 40 °C.

### MALDI TOF-MS measurement

The isolated D-arabino-1,4-lactone solution was completely dried using a rotary evaporator and a centrifugal evaporator. After drying, 50 µL of 0.01 M NaCl was added and dissolved by vortexing. 10 µL of the solution was suspended with 10 µL of 2,5-dihydroxybenzoic acid as a matrix. Measurements were performed using a matrix-assisted laser desorption/ionization time-of-flight mass spectrometer (MALDI-TOF MS, AXIMA-TOF2, SHIMADZU). 10 µL of the mixture was dropped onto a plate for MALDI TOF-MS measurement. After dropping, it was dried using a dryer. Then, the measurements were performed in positive linear mode.

### ^1^H NMR measurement

For ^1^H NMR measurement, D_2_O containing DSS as an internal standard was added to the D-arabino-1,4-lactone solution isolated above and mixed ^1^. H NMR spectra were recorded at 500 MHz using a JNM-ECP500 (JEOL). D-arabino-1,4-lactone: ^1^H NMR (500 MHz, D_2_O): δ = 3.77 (d, 1 H, J = 4.7 Hz, H-5b), 3.97–4.01 (m, 2 H, H-2, H-5a), 4.27 (d, 1 H, J = 8.9 Hz, H-2), 4.62 (dd, 1 H, J = 1.4, 1.4 Hz, H-1).

### Preparation of *Bifidobacterium* suspension

Bifidobacterial strains (*Bifidobacterium catenulatum* (*B. catenulatum*), JCM1194^T^; *Bifidobacterium pseudocatenulatum* (*B. pseudocatenulatum*), JCM1200^T^; and *Bifidobacterium breve* (*B. breve*), JCM7016) were obtained from the Microbe Division of the RIKEN BioResource Research Center, a National Research and Development Agency in Japan. Each *Bifidobacterium* was cultured as follows. First, it was anaerobically cultured at 37 °C for 24 h using modified GAM broth (NISSUI 05433) and an Anaeropack (Sugiyamagen Co., Ltd.). The culture solution was centrifuged at 4 °C, 3,500 rpm for 15 min, the supernatant was discarded, and the pellet was washed with 1 mL of physiological saline. This washing process was repeated four times, and then the suspension was adjusted to an optical density of 0.7 at 660 nm, using a medium for the sugar assimilation test.

### Preparation of *E. coli* suspension

*Escherichia coli* (*E. coli*, NBRC106373) was obtained from the NITE Biological Resource Center. *E. coli* was cultured as follows. First, *E. coli* was shake-cultured at 30 °C for 16 h using NB medium (05514, NISSUI). Then, the culture solution was centrifuged at 4 °C, 3,500 rpm for 10 min, the supernatant was discarded, and the pellet was washed with 1 mL of medium for the sugar assimilation test. This washing process was repeated 3 times, and then the *E. coli* suspension was adjusted to an optical density of 0.5 at 660 nm, using the medium for the sugar assimilation test.

### Sugar assimilation test

To prepare the medium for the sugar assimilation tests, 1 g of peptone (KISHIDA chemical), 0.5 g of sodium chloride (FUJIFILM Wako Pure Chemical Corp.), 1.2 mL of 0.2% bromothymol blue solution (FUJIFILM Wako Pure Chemical Corp.), and 1 g of yeast extract (Nacalai Tesque, Inc.) were added to 100 mL of ultrapure water. The mixture was then thoroughly mixed and sterilized using an autoclave. 30 mg each of glucose (FUJIFILM Wako Pure Chemical Corp.), sodium gluconate (FUJIFILM Wako Pure Chemical Corp.), or D-arabino-1,4-lactone (Sigma Aldrich) was added to 2.97 mL of the prepared medium for the sugar assimilation test and dissolved using a vortex. Then, 30 µL of the bacterial solution prepared above was added and suspended using a vortex. After anaerobic culture using an Anaeropack for 48 h, optical density at 660 nm was measured.

The optical density at 660 nm positively correlates with the bacterial concentration. Therefore, using the following equation, the relative value of the optical density of bacteria in the medium containing each sugar to the optical density of bacteria in the medium containing glucose was calculated as the normalized optical density. This value was used as the relative bacterial concentration.

$$\begin{aligned} \:{\text{Normalized}}\:{\text{OD}}_{{660\:{\text{nm}}}} \: & = \frac{{{\text{Optical}}\:{\text{density}}\:{\text{of}}\:{\text{medium}}\:{\text{with}}\:{\text{each}}\:{\text{sugar}}\:\left( {{\text{OD}}_{{660\:{\text{nm}}}} } \right)}}{{{\text{Optical}}\:{\text{density}}\:{\text{of}}\:{\text{medium}}\:{\text{with}}\:{\text{glucose}}\:\left( {{\text{OD}}_{{660\:{\text{nm}}}} } \right)}} \\ \: & = {\text{relative}}\:{\text{bacterial}}\:{\text{concentration}} \\ \end{aligned}$$

### Quantification of lactic acid

The bacterial culture was subjected to anaerobic cultivation for 48 h and heated at 80 °C for 15 min. After heating, the culture was centrifuged at 12,000 × g for 10 min and the supernatant was collected. The supernatant was used to quantify the lactic acid. The F-kit D-lactic acid/L-lactic acid (J. K. International) was employed for the quantitative analysis of lactic acid.

## Results and discussion

### TiO_2_ treatment of D-fructose and product analysis

**Fig. 1:**
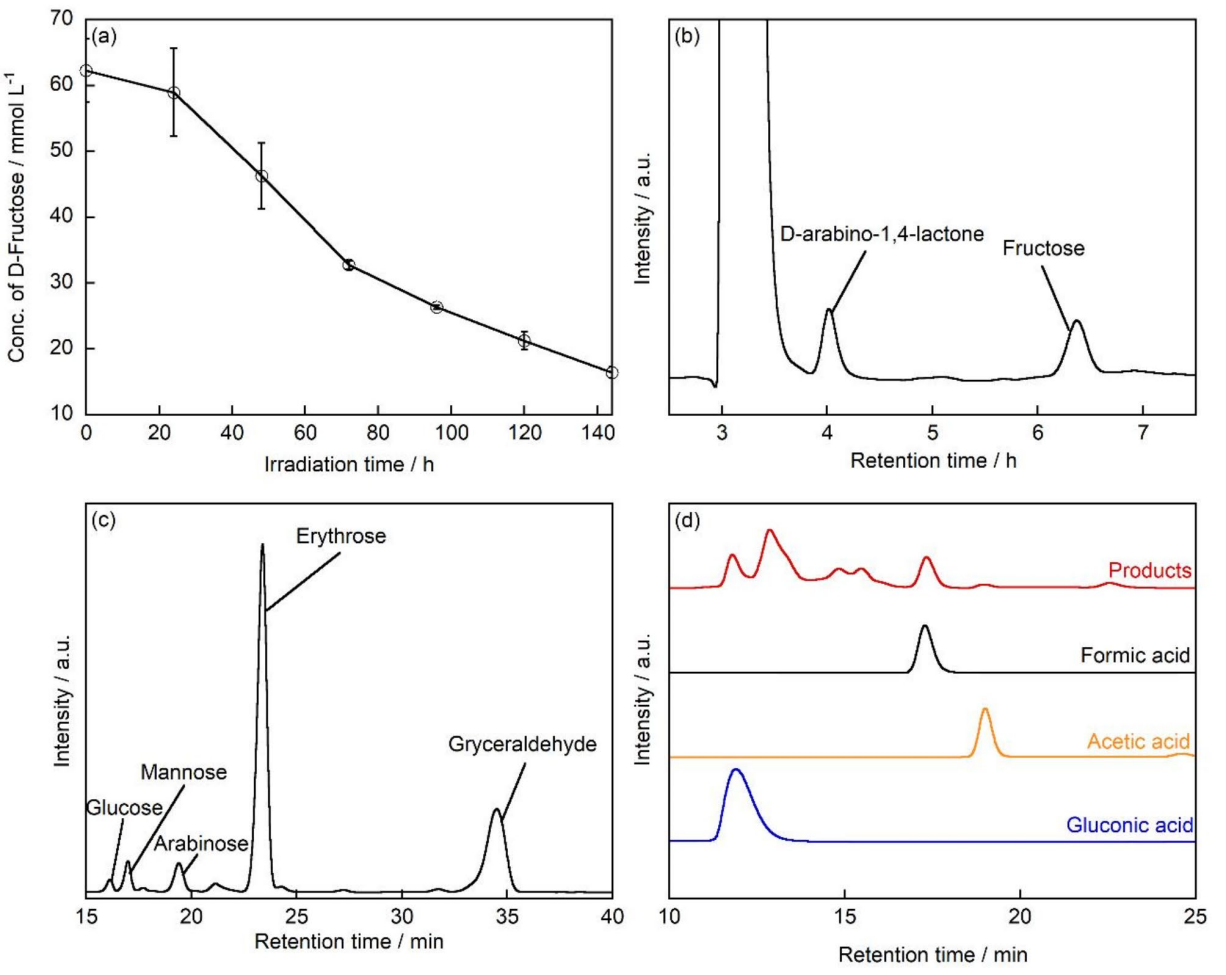
(**a**) D-fructose concentration as a function of UV irradiation time. HPLC chromatograms for obtained sample after TiO_2_ treatment of D-fructose after 144 h of UV irradiation: (**b**) D-fructose: 60 mmol L^− 1^, TiO_2_: 35 mg, UV light: 10 mW cm^− 2^, temperature: 25 ºC, Column: Sugar-D, detector: RI; (**c**) Products are functionalized with ABEE. The unreacted ABEE-labeled fructose derivative is not displayed in the chromatogram due to its short retention time in the HPLC analysis. Column: CAPCELL PAK C18, detector: UV-VIS; (**d**) Column: Rezex ROA-Organic Acid H+ (8%), detector: UV-VIS.

TiO_2_ was used to treat an aqueous solution of D-fructose under UV irradiation at room temperature in air. Figure 1a shows the concentration of D-fructose as a function of UV irradiation time. The concentration of D-fructose gradually decreased from 60 mmol L^− 1^ before UV irradiation to 16.4 mmol L^− 1^ after 144 h of UV irradiation. This suggests that D-fructose was decomposed by the photocatalytic reaction of TiO_2_ under UV irradiation, resulting in a decrease in its concentration.

Figure 1b shows the chromatogram obtained from the HPLC analysis with an RI detector for the products formed during the treatment of D-fructose with TiO_2_ after 144 h of light irradiation. The peaks with retention time (R.T.) of 6.4 min and 3.9 min corresponded to those of D-fructose and D-arabino-1,4-lactone standards, respectively. There is a larger peak observed at R.T.=3.3 min which exceeds the intensities of both D-fructose and D-arabino-1,4-lactone peaks. Since this peak was detected prior to initiating the photocatalytic reaction, it can be suggested that it does not originate from any reaction products. In HPLC analysis, such peaks are typically attributed to system-related phenomena, such as dissolved gases present in the carrier solvent. D-arabino-1,4-lactone was further isolated and measured by MALDI TOF MS, and an ion at *m/z* = 171 was detected (Fig. S1), consistent with the molecular weight of D-arabino-1,4-lactone when Na was added to D-arabino-1,4-lactone. D-arabino-1,4-lactone was further measured by ^1^H NMR. The results showed that the ^1^H NMR spectrum of D-arabino-1,4-lactone matched that of the standard compound (Fig. S2). Therefore, it is suggested that D-arabino-1,4-lactone was produced when D-fructose was decomposed by the photocatalytic reaction of TiO_2_ under UV irradiation. The other products generated after the treatment of D-fructose with TiO_2_ after 144 h of light irradiation were then analyzed by LC/MS. Figure 1c shows the HPLC chromatogram of the ABEE-labeled products. The peaks at R.T. = 16.0, 16.8, 19.2, 23.1, and 34.0 min corresponded to the R.T. of the standard compounds of glucose, mannose, arabinose, erythrose, and glyceraldehyde, respectively. Furthermore, ions with *m/z* = 330, 330, 300, 270, and 240 were detected (Fig. S3a-e), which matched the molecular weights of each sugar when ABEE-derivatized and protonated. Notably, glucose and mannose were also confirmed by HPLC before the photocatalytic reaction (Fig. S3f), suggesting that they were not produced by the photocatalytic reaction. It is known that glucose and mannose can be produced in aqueous solutions through the isomerization of D-fructose^35^. Therefore, it is considered that a small portion of the D-fructose may have been converted into these sugars via isomerization.

Next, to confirm whether aldehydes were produced after TiO_2_ treatment of D-fructose under UV irradiation, the DNPH-labeled samples were analyzed by LCMS. As a result, the R.T. of the detected peak matched the R.T. of the formaldehyde standard (Fig. S4a). Furthermore, ions with *m/z* = 209 were detected from the formaldehyde peak, which corresponded to the molecular weight of formaldehyde when DNPH-labeled and deprotonated (Fig. S4b). Additionally, the organic acids generated after the treatment of D-fructose with TiO_2_ after 144 h of light irradiation were measured, the R.T. of the detected peaks matched the R.T. of the formic acid, acetic acid and gluconic acid standards (Fig. 1d). A peak at R.T.=13 min exhibits larger intensity than those of formic acid and acetic acid. This peak was observed prior to initiating the photocatalytic reaction, indicating that it does not originate from reaction products. Moreover, since the peak appeared exclusively after fructose addition, it may be attributed to residual high-concentration fructose in the system.

**Fig. 2:**
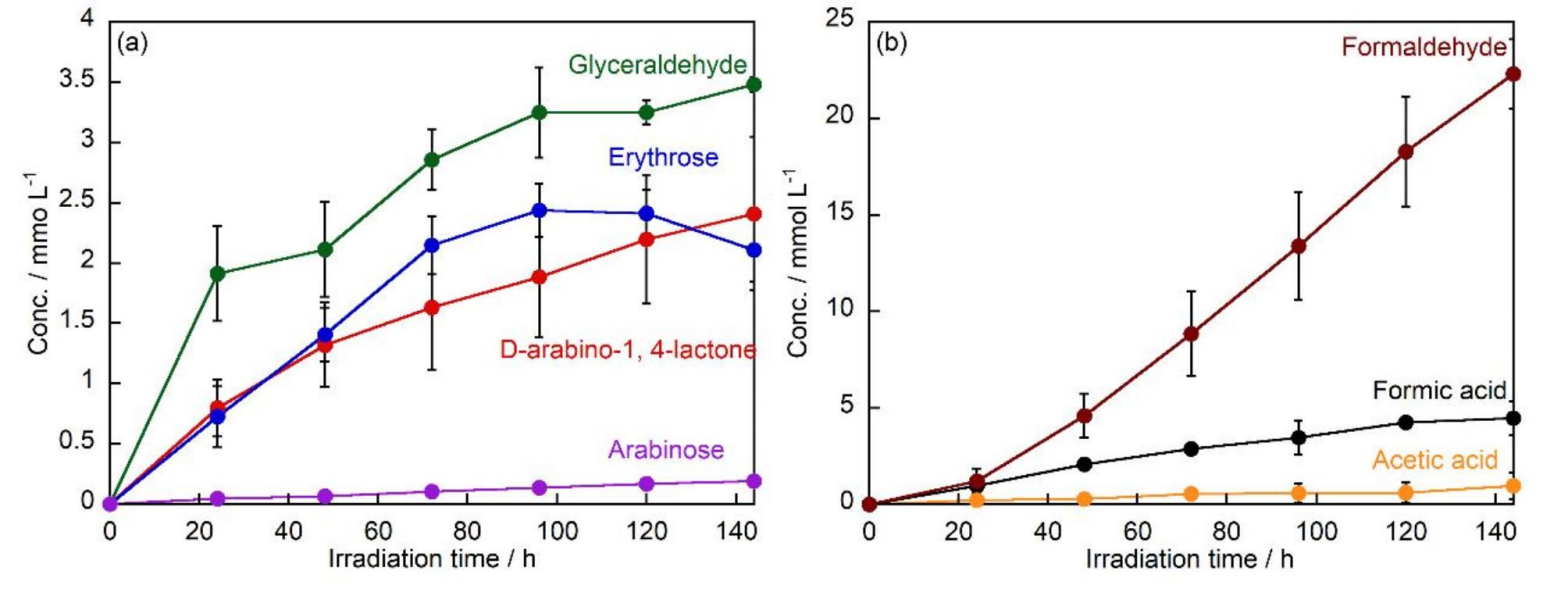
Changes in the concentrations of (**a**) D-arabino-1,4-lactone, arabinose, erythrose, and glyceraldehyde, and (**b**) formaldehyde, formic acid, and acetic acid produced by TiO_2_ treatment of D-fructose under UV irradiation.

The amount of products produced after the TiO_2_ treatment of D-fructose under UV irradiation was compared for each time (Fig. 2a). The concentrations of the arabinose and D-arabino-1,4-lactone, increased to 0.19 mmol L^− 1^ and 2.4 mmol L^− 1^ after 144 h of UV irradiation, respectively. The concentration of erythrose increased to 2.4 mmol L^− 1^ after 96 h of UV irradiation, but decreased after 96 h. After 144 h of UV irradiation, glyceraldehyde was the major product throughout all the reaction times, and the concentration of glyceraldehyde increased to 3.5 mmol L^− 1^ after 144 h of UV irradiation. The production of D-arabino-1,4-lactone from fructose may continue to increase beyond 144 h of irradiation due to the presence of residual starting material. However, as the fructose concentration decreases, the decomposition rate of D-arabino-1,4-lactone is expected to exceed its photocatalytic production rate. Consequently, a decrease in D-arabino-1,4-lactone concentration should be observed, similar to the behavior observed with erythrose.

In addition, the amounts of formaldehyde, formic acid and acetic acid formed were compared for each time (Fig. 2b). The concentration of formaldehyde increased to 22.3 mmol L^− 1^ after 144 h of UV irradiation. Formaldehyde is thought to be formed during the conversion of D-fructose to D-arabino-1,4-lactone. In addition, the oxidative degradation of glyceraldehyde to formaldehyde has been reported^36^. Since glyceraldehyde is the major product of D-fructose degradation, it is considered that a large amount of formaldehyde, its degradation product, was also produced. The organic acids, formic acid and acetic acid, increased to 4.5 mmol L^− 1^ and 0.97 mmol L^− 1^, respectively, after 144 h of UV irradiation. It has been reported that formic acid is produced during the degradation reaction of sugars^37^. Thus, the amount of formic acid produced continued to increase because formic acid is produced during the further degradation of the sugar produced by the degradation of D-fructose.

## Reaction mechanisms

On the basis of the above results, the reaction pathways by which each product was formed from D-fructose are discussed. In the TiO_2_ photocatalytic reaction of D-fructose (having six carbon atoms, C6), D-arabino-1,4-lactone and arabinose (both having five carbon atoms, C5) were formed. However, the concentration of arabinose was much lower than that of D-arabino-1,4-lactone, thus, the main C5 product obtained from D-fructose by TiO_2_ photocatalysis is D-arabino-1,4-lactone. Comparing the structures of D-fructose and D-arabino-1,4-lactone, it is assumed that D-arabino-1,4-lactone is formed by α scission (C_1_-C_2_ position cleavage) of D-fructose.

**Fig. 3:**
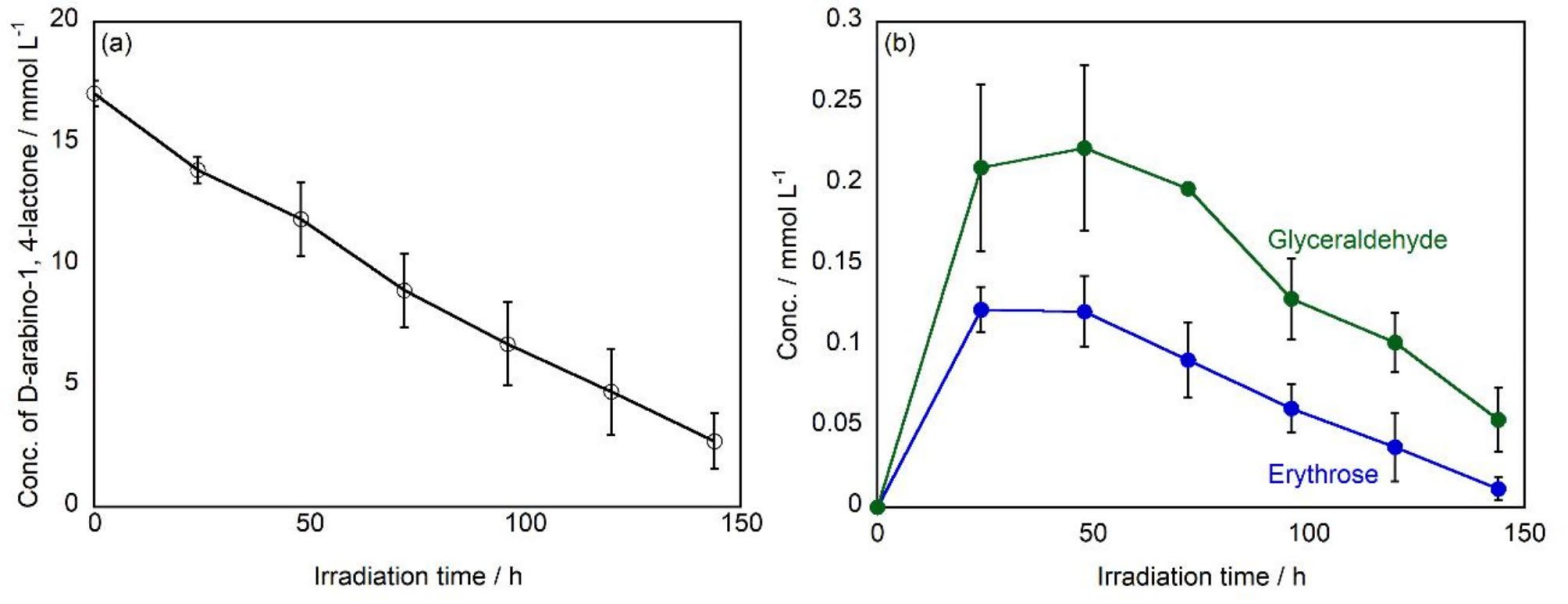
(**a**) Changes in the concentration of D-arabino-1,4-lactone, and (**b**) products as a function of UV irradiation time.

In previous reports, when glucose (C6) was decomposed using a TiO_2_ photocatalyst, arabinose (C5), erythrose (C4), and glyceraldehyde (C3) are produced, with the order of yield being C5 > C4 > C3^38^. This is because glucose undergoes a stepwise reaction, first being degraded to arabinose, and then arabinose being further degraded to erythrose (C6◊C5◊C4◊C3). However, in this study, when D-fructose (C6) was decomposed, glyceraldehyde (C3) was produced in the greatest amount, followed by erythrose (C4), and arabinose (C5) had the lowest yield (C3 > C4 > C5). This result suggests that erythrose and glyceraldehyde are produced not only via the aforementioned stepwise reaction but also through different reaction pathways involving C-C bond cleavage. On the other hand, to further investigate the reaction mechanism, we studied how D-arabino-1,4-lactone (C5), produced from D-fructose (C6), is further decomposed by the TiO_2_ photocatalytic reaction (Fig. 3a). As a result, the concentration of D-arabino-1,4-lactone gradually decreased and was completely degraded after 144 h using TiO_2_ under UV irradiation. Figure 3b shows a comparison of the amount of each product formed by the photocatalytic degradation of D-arabino-1,4-lactone. The formation of erythrose and glyceraldehyde was confirmed. Glyceraldehyde was the main product throughout all reaction times, and the concentration increased and reached to 0.22 mmol L^− 1^ after 48 h of UV irradiation, followed by a gradual decrease, ultimately declining to 0.05 mmol L^− 1^ at 144 h. The concentration of erythrose exhibited a maximum of 0.12 mmol L^− 1^ at 24 h of UV irradiation, after which it decreased continuously, reaching 0.01 mmol L^− 1^ at 96 h.

Therefore, when D-arabino-1,4-lactone was decomposed using a TiO_2_ photocatalyst, more glyceraldehyde (C3) was produced than erythrose (C4). This suggests that, similar to the case with D-fructose, both erythrose (C4) and glyceraldehyde (C3) are generated not only through the stepwise reaction from D-arabino-1,4-lactone (C5) but also via reaction pathways involving C-C bond cleavage.

**Fig. 4:**
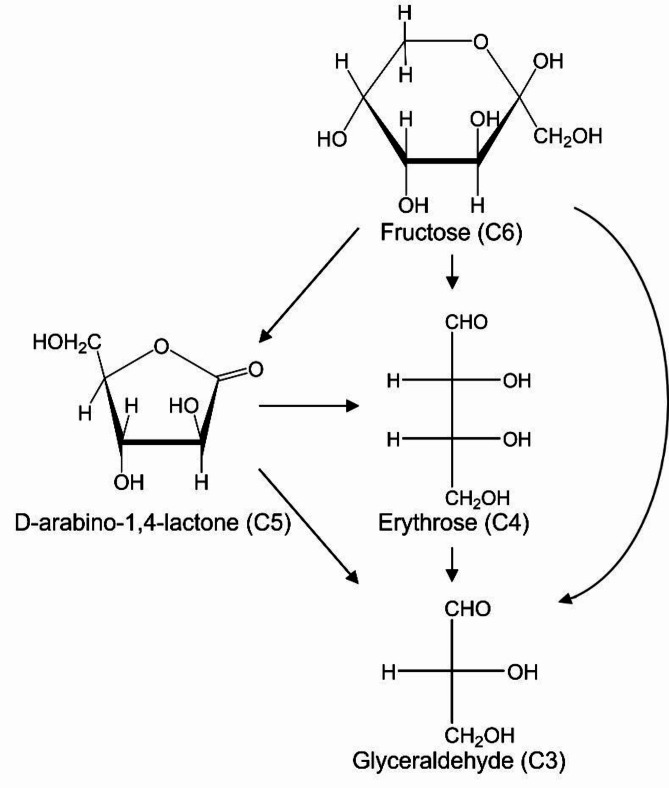
Presumed reaction mechanism of decomposition of D-fructose and generation of products using TiO_2_ photocatalyst under light irradiation.

From the above results, not only D-arabino-1,4-lactone as a lactone, but also erythrose and glyceraldehyde were produced by the TiO_2_ photocatalytic reaction of D-fructose. The reaction pathway suggests that D-arabino-1,4-lactone is formed through the degradation reaction of D-fructose, while erythrose and glyceraldehyde are mainly produced by C-C bond cleavage. Additionally, the decomposition of D-arabino-1,4-lactone also resulted in the formation of erythrose and glyceraldehyde. The reaction pathways are summarized in Fig. 4. Erythrose and glyceraldehyde are rare sugars. In recent years, rare sugars have been expected to be used as pharmaceutical agents and functional food additives^39–41^. However, they are difficult to obtain because they rarely exist in nature. It has been revealed that D-arabino-1,4-lactone can also be used as a raw material to produce the rare sugars erythrose and glyceraldehyde.

## Biological activity tests of D-arabino-1,4-lactone

In this study, we investigated D-arabino-1,4-lactone, a lactone newly confirmed to be produced by the photocatalytic decomposition of D-fructose. D-arabino-1,4-lactone has been reported to be the substrate for arabino-1,4-lactone oxidase, an enzyme involved in the final stage of ascorbic acid biosynthesis^42^. This enzyme catalyzes the conversion of D-arabino-1,4-lactone to ascorbic acid. Ascorbic acid is an important antioxidant for many organisms. D-arabino-1,4-lactone can be a raw material for synthesizing ascorbic acid. However, this compound has been reported to be produced in limited amounts, and its biological activity has not been extensively studied. Therefore, we decided to explore the biological activity of D-arabino-1,4-lactone. Gluconolactone has been reported to be assimilated by *Bifidobacterium* and is expected to be used as a prebiotic^43^. We hypothesized that D-arabino-1,4-lactone may also be assimilated by *Bifidobacterium*. *Bifidobacterium* has also been reported to have effects such as the production of B vitamins^44^, the suppression of the formation of carcinogenic substances and intestinal putrefactive products^45,46^, the prevention of constipation^47^, and the enhancement of the body’s immune system^48^. In particular, breastfed infants develop an intestinal flora composed almost entirely of *Bifidobacterium* at an early stage. The formation of this specific intestinal flora is thought to play a crucial role in preventing infectious diseases in infants^49^. In this study, we used three species of *Bifidobacterium* for the sugar assimilation test: *B. catenulatum* and *B. pseudocatenulatum*, which are known to have growth-promoting effects with sodium gluconate^50^, and *B. breve*, which are resistant to gastric acid and can easily reach the intestine alive^51^ and have also been reported to have anti-obesity effects^52^. *B. infantis* and *B. breve* are known as important *Bifidobacterium* species that have been reported to become dominant in the intestines of breastfed infants due to priority effects. Among these, *B. infantis* possesses the ability to degrade all human milk oligosaccharides (HMOs)^53^. In contrast, despite its very low HMO utilization capacity, *B. breve* has been reported to become dominant in the intestines of many breastfed infants^53^. Additionally, *B. breve* has been widely reported to be effective from a probiotics perspective^54^. For these reasons, we decided to investigate the effects on *B. breve*. For comparison, we also used *E. coli*.

**Fig. 5:**
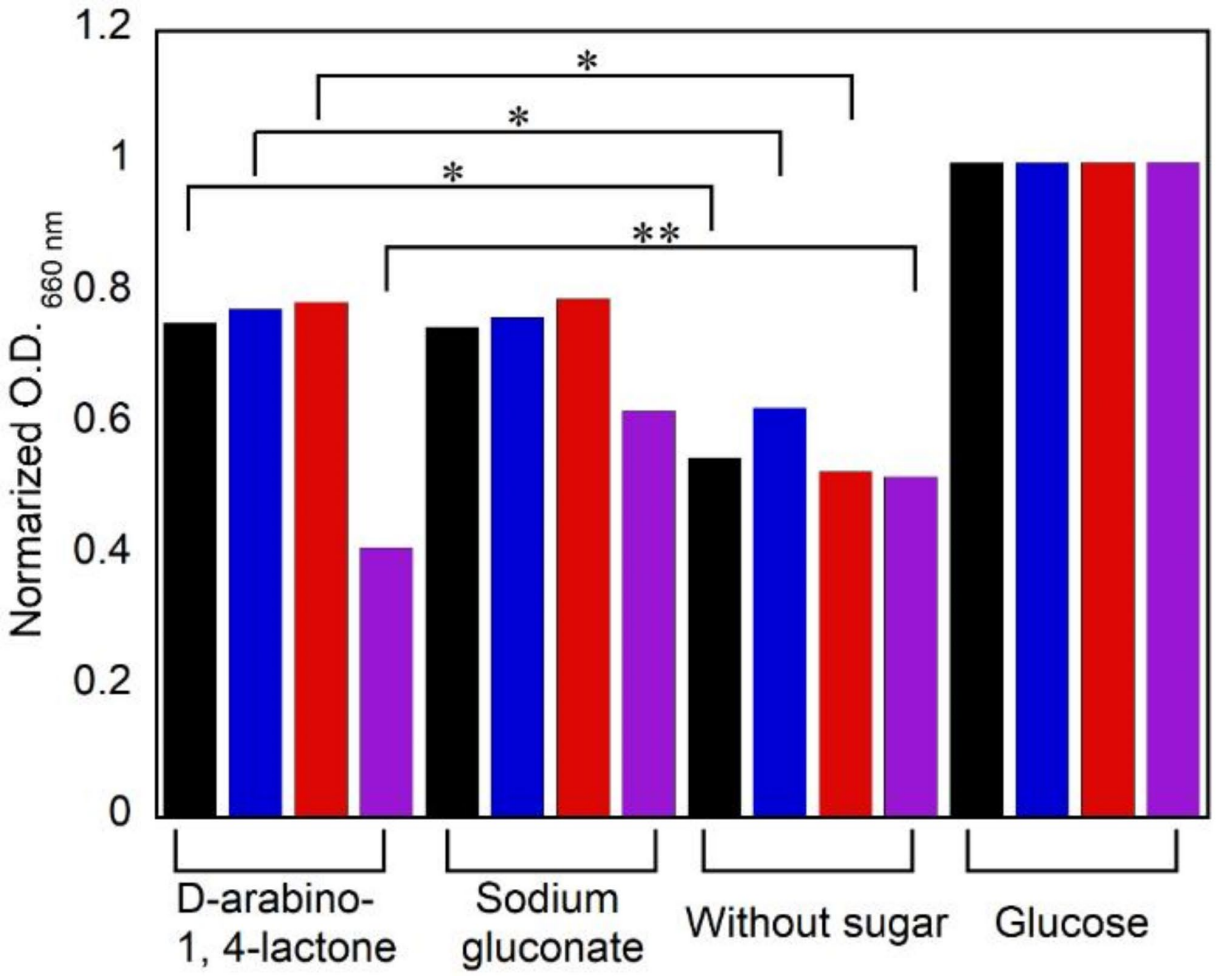
Assimilation test by bacteria using each sugar after 48 h incubation. Black: *B. catenulatum*, blue: *B. pseudocatenulatum*, red: *B. breve*, purple: *E. coli* (*n* = 3, *p < 0.05, **p < 0.01).

The relative value of the optical density of bacteria in the medium containing each sugar to the optical density of bacteria in the medium containing glucose was calculated as the normalized optical density. Additionally, since the optical density positively correlates with the bacterial concentration, the normalized optical density represents the relative value of the bacterial concentration in the medium under each condition compared to the bacterial concentration cultured in the medium containing glucose. The addition of sodium gluconate to the medium served as a positive control, as this compound has been reported to be assimilated by *Bifidobacterium*^50^, and the case where no sugar was added to the medium was used as a negative control. For *B. catenulatum*, *B. pseudocatenulatum*, and *B. breve*, the relative bacterial concentrations were as follows: 0.76, 0.78, and 0.79, respectively, when D-arabino-1,4-lactone was added; 0.75, 0.76, and 0.79, respectively, when sodium gluconate was added; and 0.55, 0.63, and 0.53, respectively, when no sugar was added (Fig. 5). From the above results, although the relative values of the bacterial concentration when D-arabino-1,4-lactone was added were lower than 1 in all *Bifidobacterium* species, indicating a lower bacterial concentration than when only glucose was used, a significant increase in the bacterial concentration was observed compared with that when no sugar was added (*p* < 0.05). Additionally, these values were similar to those of sodium gluconate, and no significant differences were observed. These results confirm that *B. catenulatum*, *B. pseudocatenulatum*, and *B. breve* can proliferate in the presence of D-arabino-1,4-lactone to a similar extent as in the presence of sodium gluconate.

On the other hand, we also conducted a sugar assimilation test using *E. coli*, which can have a negative impact on the human body if it is present in excess in the intestine. When *E. coli* was used, the relative values of the bacterial concentration were 0.41 when D-arabino-1,4-lactone was added, 0.62 when sodium gluconate was added, and 0.52 when no sugar was added. When D-arabino-1,4-lactone was used, the relative value of the bacterial concentration significantly decreased from 1, and there were significant differences compared to when sodium gluconate was used or when no sugar was added (*p* < 0.01). In glucose-free medium, D-arabino-1,4-lactone was found to inhibit the growth of *E. coli.* Since *E. coli* lacks D-arabinono-1,4-lactone oxidase^55^, the D-arabino-1,4-lactone added to the medium remains unmetabolized. This D-arabino-1,4-lactone exists in equilibrium with arabonic acid^56^, which causes the pH of the medium to decrease. Indeed, after 48 h of cultivation, the pH of the medium decreased to 4, which significantly deviates from the optimal pH range for *E. coli* (7.0-7.5)^57^. This pH reduction is considered to be the primary factor responsible for the inhibition of *E. coli* growth^58^.

*Bifidobacterium* produces lactic acid during the process of assimilating sugars such as glucose^59^. Therefore, lactic acid was measured to investigate whether the sugars added to the medium were assimilated. Table 1 shows the lactic acid concentration in the medium when the sugar assimilation test was conducted using *B. breve*, which had the highest relative bacterial concentration in the presence of D-arabino-1,4-lactone. The D-lactic acid concentrations were 1.57, 0.871, 18.6, and 17.4 mmol/L, and the L-lactic acid concentrations were 0.0610, 0.154, 0.466, and 1.40 mmol/L, respectively, for media containing D-arabino-1,4-lactone, no sugar, sodium gluconate, or glucose. The total lactic acid concentrations were 1.63, 1.02, 18.8, and 19.1 mmol/L for media containing D-arabino-1,4-lactone, no sugar, sodium gluconate, or glucose, respectively. The amount of lactic acid produced with D-arabino-1,4-lactone was lower than that produced with sodium gluconate or glucose, which served as positive controls. Although the amount of L-lactic acid was lower with D-arabino-1,4-lactone than without sugar, the total amount of D, L-lactic acid was greater with D-arabino-1,4-lactone. These results suggest that *B. breve* assimilates D-arabino-1,4-lactone.

**Table 1:** Concentration of lactic acid in medium after assimilation test using *B. breve* (mmol L^− 1^).

	D-arabino-1,4-lactone	No sugar	Sodium gluconate	Glucose
D-lactic acid	1.57	0.871	18.6	17.4
L-lactic acid	0.0610	0.154	0.466	1.40
Total	1.63	1.02	18.8	19.1

## Conclusions

In this study, the production of D-arabino-1,4-lactone, as a lactone, from D-fructose using TiO_2_ photocatalyst was confirmed. The reaction mechanism involves α scission (C_1_-C_2_ position cleavage) of D-fructose to produce D-arabino-1,4-lactone, as well as degradation and C-C bond cleavage of D-fructose and D-arabino-1,4-lactone to produce erythrose and glyceraldehyde. The results of the sugar assimilation test and lactic acid measurement using D-arabino-1,4-lactone revealed that *Bifidobacterium* proliferates by assimilating D-arabino-1,4-lactone. Additionally, the growth of *E. coli* was inhibited by adding D-arabino-1,4-lactone. Therefore, it can be expected that D-arabino-1,4-lactone can be used for the growth of *Bifidobacterium* and as a new food additive.

## Electronic supplementary material

Below is the link to the electronic supplementary material.

Supplementary Material 1

## Data Availability

All data included in this study is available upon request by contact with the corresponding author.
