# Supplementary material for: Photocatalytic production and biological activity of D-arabino-1,4-lactone from D-fructose
Source: Sci Rep. 2025 Jan 11;15:1708. doi: 10.1038/s41598-024-84921-z (PMC11724892; doi:10.1038/s41598-024-84921-z)
Supplement: Supplementary file 1 — Supplementary Material 1 [file 41598_2024_84921_MOESM1_ESM.docx]

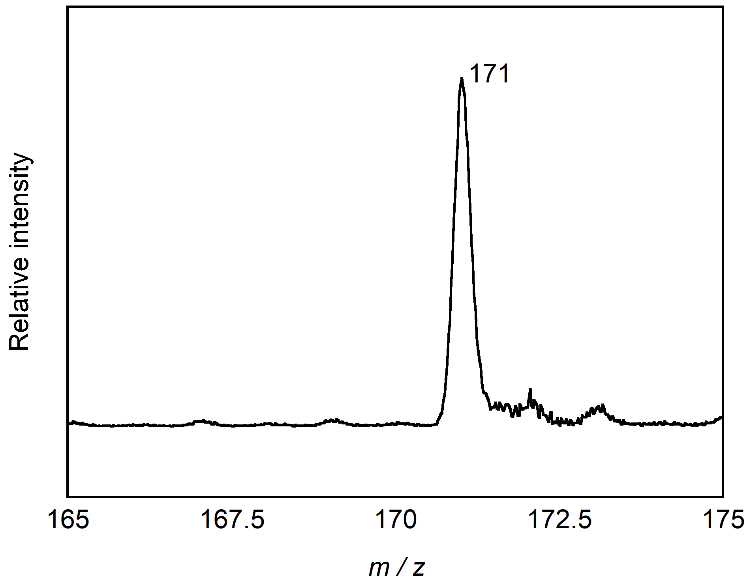


**Fig. S1.** MALDI TOF MS spectrum for obtained sample after TiO_2_ treatment of fructose under UV irradiation. 60 mmol L^-1^, TiO_2_ photocatalyst: 35 mg, UV light: 10 mW cm^-2^, temperature: 25ºC.


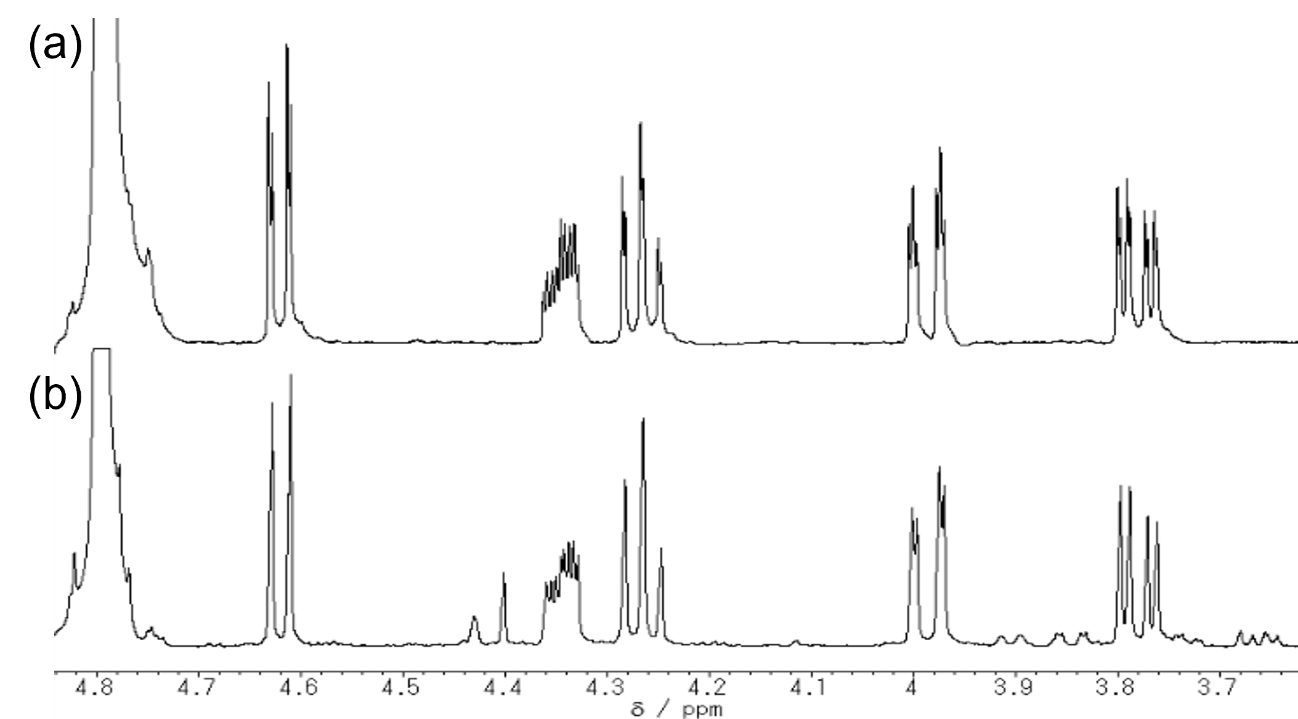


**Fig. S2.** ^1^H NMR spectra of (a) standard and (b) obtained D-arabino-1,4-lactone. Fructose: 60 mmol L^-1^, TiO_2_ photocatalyst: 35 mg, UV light: 10 mW cm^-2^, temperature: 25ºC.


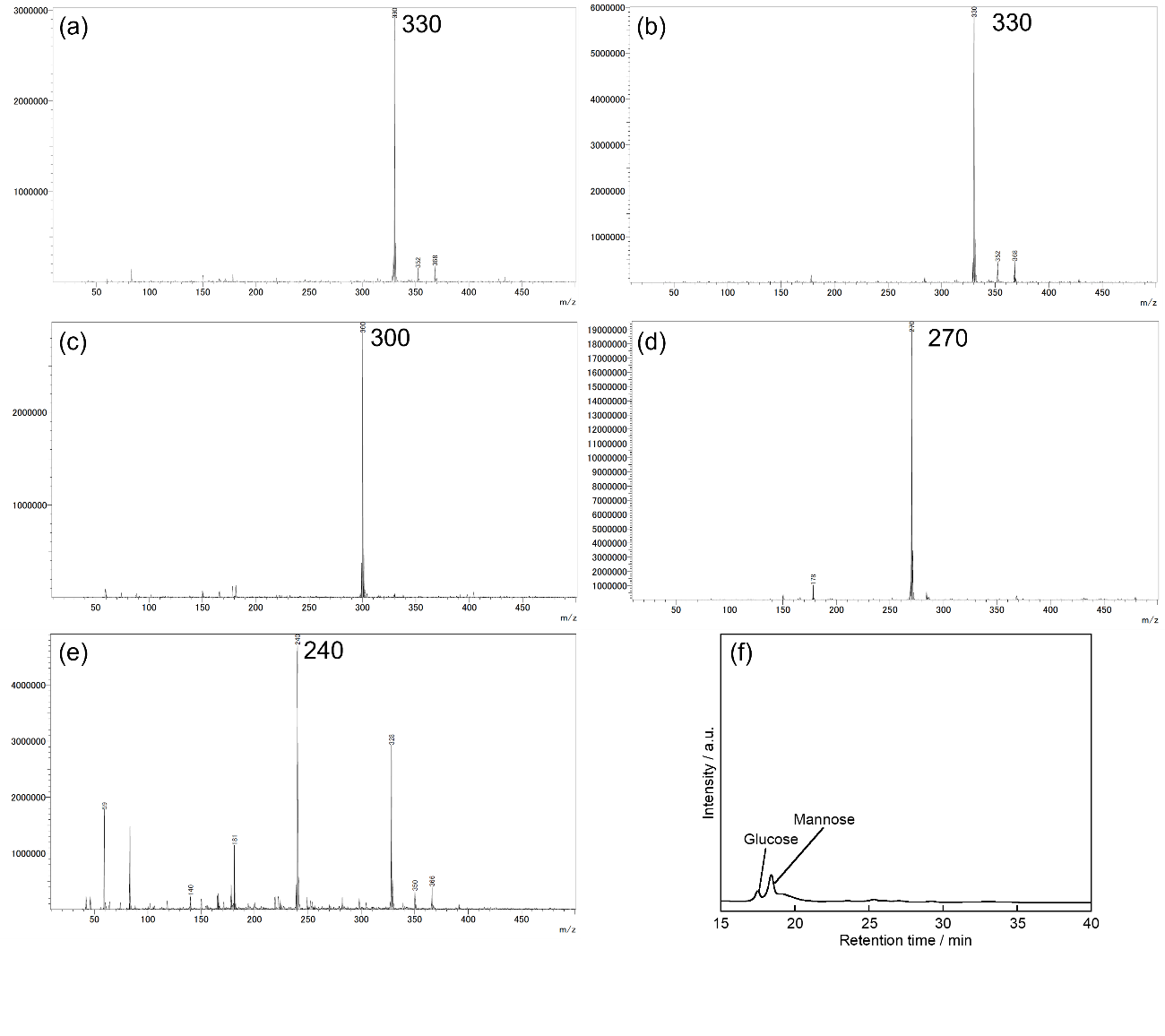


**Fig. S3.** LC/MS spectra for obtained (a) glucose, (b) mannose, (c) arabinose, (d) erythrose, (e) glyceraldehyde after TiO_2_ treatment of fructose under UV irradiation. (f) HPLC chromatogram for obtained sample before TiO_2_ treatment of fructose under UV irradiation. Products are functionalized with ABEE. Fructose: 60 mmol L^-1^, TiO_2_ photocatalyst: 35 mg, UV light: 10 mW cm^-2^, temperature: 25ºC, column: CAPCELL PAK C18, detector: UV-VIS.


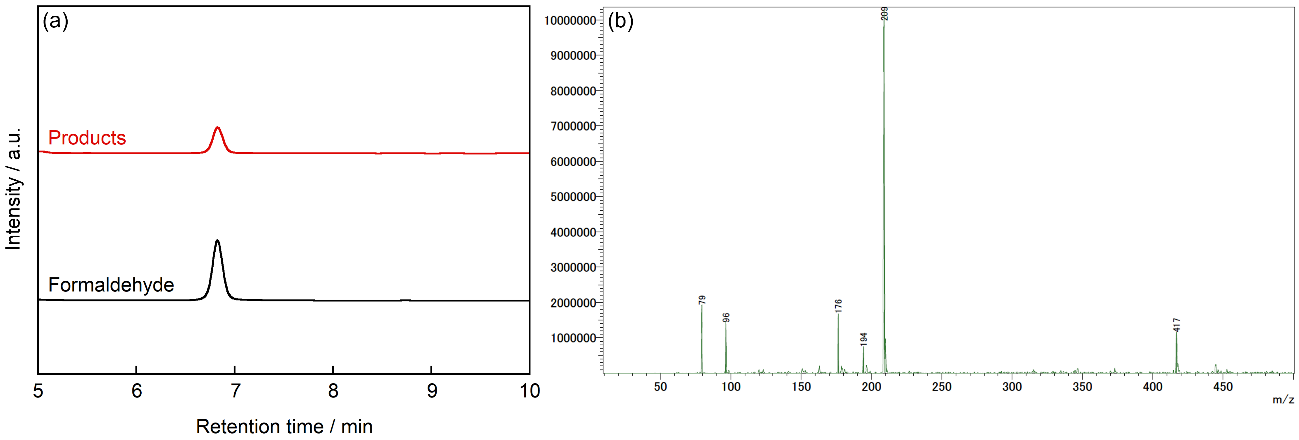


**Fig. S4.** (a) HPLC chromatogram for obtained sample after TiO_2_ treatment of fructose under UV irradiation. (b) LC/MS spectrum for obtained sample after TiO_2_ treatment of fructose under UV irradiation. Products are functionalized with DNPH. 60 mmol L^-1^, TiO_2_ photocatalyst: 35 mg, UV light: 10 mW cm^-2^, temperature: 25ºC, column: Luna C18(2), detector: UV-VIS.
